# Secondary Osteoporosis and Metabolic Bone Diseases

**DOI:** 10.3390/jcm11092382

**Published:** 2022-04-24

**Authors:** Mahmoud M. Sobh, Mohamed Abdalbary, Sherouk Elnagar, Eman Nagy, Nehal Elshabrawy, Mostafa Abdelsalam, Kamyar Asadipooya, Amr El-Husseini

**Affiliations:** 1 Mansoura Nephrology and Dialysis Unit, Mansoura University, Mansoura 35516, Egypt; sobh_92@hotmail.com (M.M.S.); mamdouh.abdalbary@uky.edu (M.A.); sherouk43@gmail.com (S.E.); emannagy@mans.edu.eg (E.N.); dr.nehalelshabrawy@mans.edu.eg (N.E.); moustafaabdelsalam@yahoo.com (M.A.); 2 Division of Nephrology, Bone and Mineral Metabolism, University of Kentucky, Lexington, KY 40506, USA; 3 Division of Endocrinology, University of Kentucky, Lexington, KY 40506, USA; kas224@uky.edu

**Keywords:** bone loss, fracture, bone mineral density, causes, management

## Abstract

Fragility fracture is a worldwide problem and a main cause of disability and impaired quality of life. It is primarily caused by osteoporosis, characterized by impaired bone quantity and or quality. Proper diagnosis of osteoporosis is essential for prevention of fragility fractures. Osteoporosis can be primary in postmenopausal women because of estrogen deficiency. Secondary forms of osteoporosis are not uncommon in both men and women. Most systemic illnesses and organ dysfunction can lead to osteoporosis. The kidney plays a crucial role in maintaining physiological bone homeostasis by controlling minerals, electrolytes, acid-base, vitamin D and parathyroid function. Chronic kidney disease with its uremic milieu disturbs this balance, leading to renal osteodystrophy. Diabetes mellitus represents the most common secondary cause of osteoporosis. Thyroid and parathyroid disorders can dysregulate the osteoblast/osteoclast functions. Gastrointestinal disorders, malnutrition and malabsorption can result in mineral and vitamin D deficiencies and bone loss. Patients with chronic liver disease have a higher risk of fracture due to hepatic osteodystrophy. Proinflammatory cytokines in infectious, autoimmune, and hematological disorders can stimulate osteoclastogenesis, leading to osteoporosis. Moreover, drug-induced osteoporosis is not uncommon. In this review, we focus on causes, pathogenesis, and management of secondary osteoporosis.

## 1. Introduction

**Figure 1:**
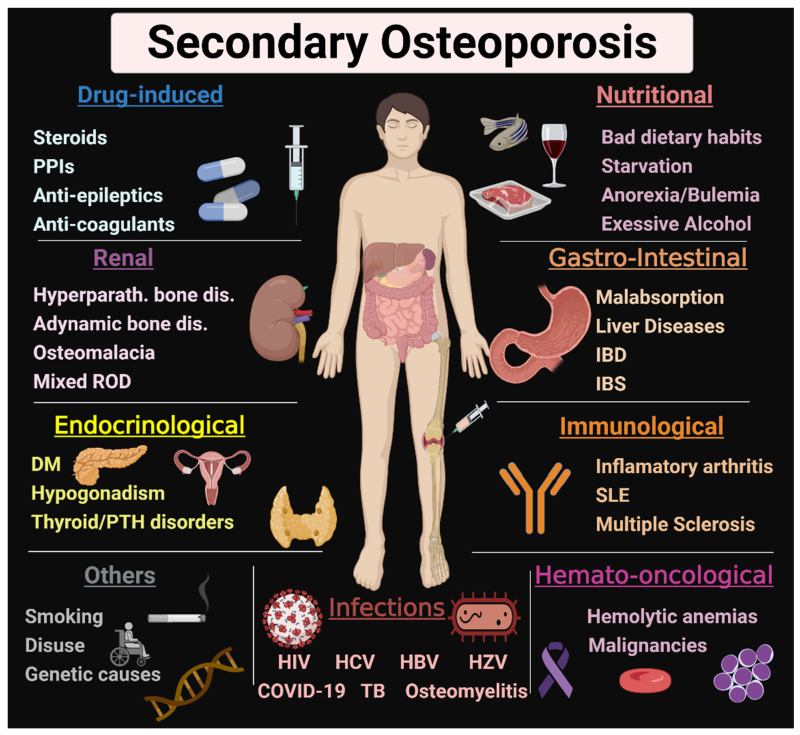
Causes of secondary osteoporosis. Various causes of secondary osteoporosis are illustrated in this figure. They include ROD, DM, thyroid and parathyroid disorders, malabsorption, IBD, IBS, nutritional causes, drug-induced, infections, anemia, malignancies, inflammatory arthritis, SLE, smoking, and genetic causes. PPIs: proton pump inhibitors, ROD: renal osteodystrophy, DM: diabetes mellitus, PTH: parathyroid, IBD: inflammatory bowel disease, IBS: irritable bowel syndrome, SLE: systemic lupus erythematosus. HIV: human immunodeficiency virus, HCV: hepatitis C virus, HBV: hepatitis B virus, HZV: herpes zoster virus, TB: tuberculosis. This Figure was created with BioRender.com (accessed on 1 February 2022).

**Figure 2:**
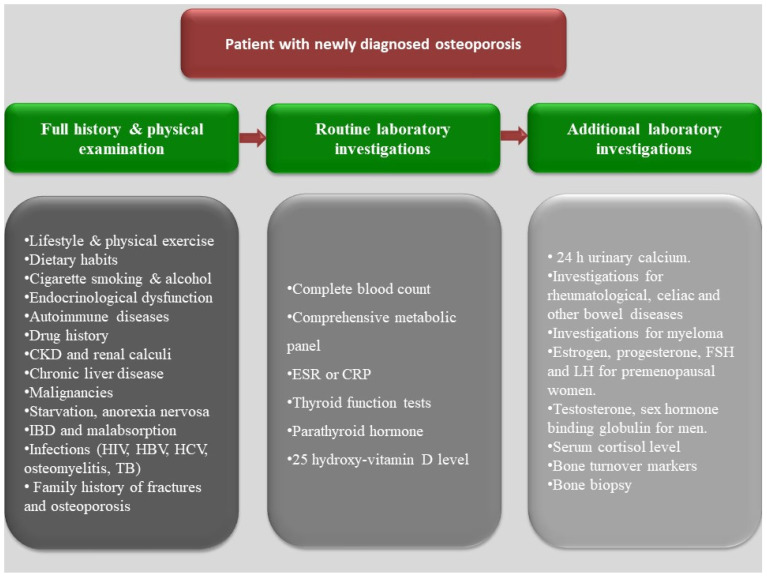
Pragmatic diagnostic approach for newly diagnosed patients with osteoporosis. A systematic approach for the analysis and detection of a secondary cause of osteoporosis is recommended for all patients with a new diagnosis of osteoporosis. A full history and physical examination followed by a routine laboratory investigation for the most common and simple underlying causes of osteoporosis are required for most cases. Some additional investigation may be considered after routine lab for the suspected cases. CKD, chronic kidney disease, CRP: C-reactive protein; ESR: erythrocyte sedimentation rate; IBD: inflammatory bowel diseases; HCV: hepatitis C virus; HBV: hepatitis B virus; HIV: human immunodeficiency virus; TB: tuberculosis; FSH: follicle stimulating hormone; LH: luteinizing hormone.

**Figure 3:**
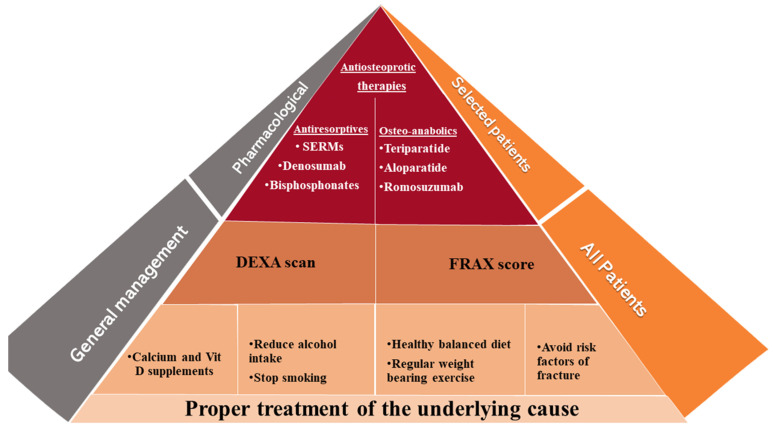
Approach for prevention and management of secondary osteoporosis. Correction of the underlying causes of secondary osteoporosis is the cornerstone of prevention and treatment. All patients can benefit from non-pharmacological intervention, DEXA scan and assessment of fracture risk. Anti-osteoporotic medications (antiresorptives and osteoanabolics) can be used in selected cases with high fracture risk. DEXA: dual-energy X-ray absorptiometry, FRAX: fracture-risk algorithm, SERM: Selective estrogen receptor modulators.

**Figure 4:**
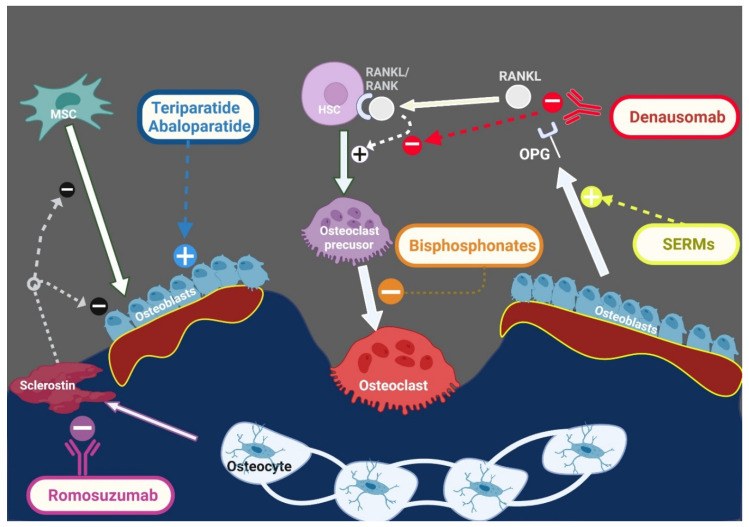
Mechanism of action of common antiosteoporotic medications. Antiosteoporotic medications can be divided into two main categories: 1. Antiresorptives “on the right side” act mainly by inhibiting osteoclasts. Bisphosphonates act by inhibiting osteoclast differentiation from osteoclast precursors. The monoclonal antibody “denausumab” inhibits osteoclast differentiation by binding to RANKL, preventing its interaction with RANK. SERMs increase OPG production, thus inhibiting osteoclastogenesis. 2. Osteoanabolics “on the left side” stimulate bone formation via activation of PTH (teriparatide) or PTH-related peptide (abaloparatide) receptors. Romosuzumab is an anti-sclerostin monoclonal antibody. Thus, it stimulates osteoblast differentiation and function. MSC: mesenchymal stem cells, HSC: hematopoietic stem cells, SERMs: selective estrogen receptor modulators, OPG: osteoprotegerin, RANK: Receptor activator of nuclear factor κ B, RANKL: receptor activator of nuclear factor kappa-Β ligand. this figure was created with BioRender.com (accessed on 1 February 2022).

Osteoporosis is a condition characterized by bone fragility, secondary to either low bone mineral density (BMD) and/or microarchitectural deterioration that increases fracture risk. Postmenopausal estrogen deficiency is the primary cause of osteoporosis. In addition to postmenopausal women with primary osteoporosis (postmenopausal or age-related), more than half of perimenopausal and postmenopausal women referred to an osteoporosis center had one or more risk factors of secondary osteoporosis [1]. A fracture risk assessment tool (FRAX) helps to estimate the 10-year fracture risk by using clinical and radiological data. These clinical data include some, but not all, secondary causes of osteoporosis, such as smoking, excessive alcohol intake, type I diabetes mellitus, hyperthyroidism, chronic liver disease, and malnutrition [2]. Various secondary causes of osteoporosis are mentioned in Figure 1. Patients with newly diagnosed osteoporosis should be thoroughly evaluated including their history, a physical examination, and routine laboratory testing for detection of secondary causes. A systematic approach for detection of the underlying causes is illustrated in Figure 2. The management approach of patients with secondary osteoporosis is summarized in Figure 3. Proper recognition of the etiology of osteoporosis is an essential step in improving bone health, preventing further bone loss. Those patients can benefit from balanced nutrition, physical exercise, and avoiding long term glucocorticoid usage and other drugs that have negative impact on bone health. Using antiosteoporotic therapies in patients with high risk of fractures is recommended; the mechanism of action of the commonly used antiosteoporotic medications are illustrated in Figure 4. This article comprehensively discusses epidemiology, the various causes and pathogenesis of secondary osteoporosis. This topic not only covers the bone quantity problem but focuses on quality as well. Furthermore, the up-to-date management of secondary osteoporosis is thoroughly discussed.

## 2. Renal Causes

Chronic kidney disease (CKD) is a well-established risk factor for bone loss [3]. The incidence of bone loss and fracture risk increases with decline in kidney function. Osteoporosis was reported in up to 32% of CKD patients, while osteopenia was found in about half [3,4,5,6]. However, the magnitude of the problem might be higher for various reasons. First, there is a high prevalence of vascular calcification in CKD patients, which results in a higher estimation of vertebral bone mass by DXA [7]. Second, CKD patients do not have a bone mass/quantity problem only, but a bone quality disorder as well [8]. Third, there is underutilization of osteoporosis diagnostic tools in CKD patients, despite the KDIGO recommendations. Up to 30–50% of fractured CKD patients had a T-score higher than −2.5 [9,10]. Advanced CKD patients have up to an 8-fold higher fracture risk when compared to the general population [11]. Osteoporotic fractures lead to a deleterious effect on the quality of life in CKD patients. One-year mortality after having a hip fracture is 17–27% in the general population [12,13], while it is up to 64% in patients with end-stage kidney disease (ESKD) [14,15].

Renal osteodystrophy (ROD), medication usage, hypogonadism, systemic inflammation, acidosis, and concurrent systemic illnesses contribute to bone loss in patients with CKD. Metabolic acidosis stimulates osteoclasts and induces robust bone resorption. ROD develops with early stages of CKD and progresses with further loss of kidney function [16]. There are many co-players in the pathogenesis of ROD. FGF-23, an osteocyte-secreted phosphaturic hormone, rises in early stages of CKD to prevent hyperphosphatemia [17,18]. Hyperphosphatemia occurs in late CKD stages despite increasing levels of FGF-23 due to klotho deficiency/resistance [19]. FGF-23 inhibits vitamin D activation and increases its catabolism [20,21]. Vitamin D deficiency/insufficiency, and hyperphosphatemia, contribute to secondary hyperparathyroidism in CKD patients [22,23,24,25]. Levels of sclerostin, DKK-1, and WNT pathway inhibitors increase with deterioration of kidney function [26]. They inhibit bone formation and promote low turnover bone disease [27]. On the other hand, the imbalance between osteoprotegerin (OPG) and receptor activator of nuclear factor kappa B ligand (RANKL) levels in CKD patients increases osteoclastogenesis and induces high turnover bone disease [28,29]. Moreover, disturbed gonadal hormones could be a major reason for osteoporosis. Many drugs commonly used in CKD patients such as heparin, warfarin, glucocorticoids, proton pump inhibitors, and diuretics can negatively affect bone health [30,31]. 

Many tools can be used in the diagnosis of osteoporosis in CKD patients, although there is no consensus on the optimal tool. DXA is the most widely used method. The Fracture Risk Assessment Tool (FRAX) helps to estimate the 10-year fracture risk; however, it does not include CKD as a secondary cause of osteoporosis [32]. Quantitative computed tomography (QCT) is not affected by vascular calcifications and could be a better tool, compared to DXA, especially for longitudinal follow-up and in obese patients [33]. However, its use is less common due to higher costs and radiation exposure. Both tools help to assess bone mass/quantity. On the other hand, TBS, high-resolution imaging techniques, finite element analysis, and Fourier transform infrared spectroscopy can be used in the assessment of bone quality. Bone turnover markers provide dynamic assessment of bone formation and resorption and facilitate ROD management [34]. Bone-specific alkaline phosphatase (BSAP) and intact procollagen-1 N-terminal peptide (P1NP) as bone formation markers, and tartrate-resistant acid phosphatase 5b (TRAP 5b) as a bone resorption marker are reliable in CKD patients [35]. Bone turnover markers and parathyroid hormone (PTH) do not only help to understand bone turnover status [36], but also to predict fracture risk [37,38]. Bone biopsy remains the gold standard to identify the mechanism and severity of bone loss [39]. It also helps to choose the appropriate medication, but it is limited by its invasive nature and lack of expertise. Assessment of bone histology in CKD patients should include three elements: turnover, mineralization, and volume [16,40]. Nowadays, the most common pathological findings in CKD patients are low turnover bone disease (LTBD), high turnover bone disease (HTBD), mixed ROD, while osteomalacia is less frequently seen in adults [41]. Recently published reviews have described the bone quality assessment and management in patients with CKD [7,42]. 

The primary step in osteoporosis management is to control the CKD metabolic derangements. Vitamin D deficiency, hyperphosphatemia, and hyperparathyroidism are common findings in these patients and have detrimental effects on bones. Patients should be instructed about fall risk prevention and non-pharmacological interventions to improve bone health. Smoking cessation, alcohol limitation, personalized exercise protocols, and well-balanced nutrition have a positive impact on bone, but are underutilized in CKD patients [42]. Optimizing calcium intake and the proper use of phosphate-lowering therapies, vitamin D, and calcimimetics can reduce fracture risks by improving ROD [43]. 

Determining the type of ROD and including high versus low turnover help to choose the appropriate treatment with higher efficacy and lower adverse events. Patients with HTBD are expected to benefit more from antiresorptives, e.g., bisphosphonates and denosumab, while patients with LTBD may benefit from osteoanabolics to improve bone formation. 

Despite being excreted by the kidneys, bisphosphonates can be used in mild to moderate CKD patients without major safety concerns [44]. Their use in advanced CKD patients should be cautious with a concern for CKD progression [45]. Moreover, prolonged use of bisphosphonates in patients with advanced CKD might induce LTBD and increase the risk of atypical femur fracture [46]. Denosumab has been shown to improve BMD and reduce bone turnover in CKD patients in observational studies and small randomized control trials (RCTs) [47,48]. As opposed to bisphosphonates, it is not excreted through the kidneys, however close monitoring of serum calcium and vitamin D should be conducted for the risk of hypocalcemia. 

On the other hand, osteoanabolics (teriparatide, abaloparatide, and romosozumab) have a promising role in mitigating bone loss in patients with LTBD. Teriparatide has been used in advanced CKD patients in several studies [49,50,51,52]. Abaloparatide was safe and effective in the early stages of CKD [53]. Romosozumab increased BMD in patients with mild to moderate CKD [54] and in dialysis patients [55].

## 3. Endocrinological Causes

### 3.1. Diabetes Mellitus

Diabetes is a chronic metabolic disease associated with an increased risk of fragility fracture. Adults with Type 1 diabetes mellitus (T1DM) have a greater risk of fracture, especially non-vertebral fracture, than those with type 2 diabetes (T2DM) [56,57]. Nevertheless, vertebral fractures are not uncommon and associated with increased mortality, but they are often underdiagnosed because they could be asymptomatic [58]. Diabetes can compromise bone metabolism, impair cell function or damage the extracellular matrix. This results in bone loss, alteration of bone microarchitecture, reduction of bone turnover and predisposition to low trauma fracture. The pathogenesis and risk factors of brittle bone in diabetes consist of obesity, increased insulin resistance, blood sugar disturbances, production of advanced glycation end products, muscle dysfunction, macro- and microvascular complications, and medications. Moreover, the associated comorbidities, such as thyroid disorders, gonadal dysfunction, and malabsorption may contribute to bone loss [59,60]. Notably, T1DM has been associated with reduced osteoblast activity, lower or similar BMD, and a higher risk of fracture [56,61,62,63]. Whereas T2DM is associated with an increased rate of bone loss and fracture, even with normal or high BMD [56,64]. A T-score threshold of −2.0 was suggested as a trigger for therapeutic intervention in T2DM [65]. However, the bone area at the total hip is a better surrogate for fragility fracture in elderly patients with T2DM compared to BMD [66]. 

Diabetes mainly affects bone quality, including disturbed bone material properties and increased cortical porosity, which are not measurable with BMD-DXA [59,67]. This emphasizes that bone density measurement by DXA underestimates the fracture risk in diabetic patients [68]. Trabecular bone score [69], peripheral quantitative computed tomography (pQCT), pQCT-based finite element analysis (pQCT-FEA) [70], and high resolution peripheral quantitative computed tomography (HR-pQCT) [71] are better tools to estimate fracture risk in diabetic patients. Invasive methods, such as microindentation and bone histomorphometry, are expensive and not widely available [68,72]. 

Diabetes causes skeletal fragility and applying strategies to reduce fracture is crucial. Furthermore, it seems there is a correlation between the degree of blood sugar control and the risk of fracture. [73,74]. In a large cohort study, there was a cubic relationship between HbA1c and risk of fracture [75]. Thiazolidinediones should be avoided in diabetic patients with increased bone fragility [76]. Moreover, there is growing evidence suggesting a negative outcome of sodium glucose cotransporter 2 (SGLT2) inhibitors on bone health. Alendronate use for 3 years resulted in an increase in BMD in diabetic patients with osteoporosis [77]. Anti-osteoporotic medications (mainly bisphosphonates) appear to prevent bone loss similarly in the spines of diabetic and non-diabetic individuals in a recent systematic review [78]. Use of daily subcutaneous injections of abaloparatide (80 mcg) was associated with improvement in BMD in diabetic patients [79].

### 3.2. Gonadal Disorders 

Hypogonadism is a risk factor for osteoporosis. The peak bone mass and BMD are higher in men; however, if both a man and a woman have similar BMD, the man would have a higher risk of fracture. The incidence of osteoporosis in men under the age of 70 is significantly lower compared to women because the bone loss in women occurs earlier and at a higher rate [80,81]. Testosterone replacement therapy can improve BMD but results in hypogonadal older men were inconclusive. However, the volumetric BMD and bone strength significantly improved in hypogonadal older men who received testosterone treatment for one year [82,83]. 

### 3.3. Parathyroid Disorders (Hypoparathyroidism and Primary Hyperparathyroidism) 

Hypoparathyroidism is a low bone turnover condition. The information regarding fracture risk is inconsistent [84,85,86], but patients with nonsurgical hypoparathyroidism seem to have a higher risk of vertebral fracture [86,87,88]. This could be potentially due to a longer period of bone changes in nonsurgical hypoparathyroidism compared to surgical hypoparathyroidism [86]. Therefore, we would speculate that the higher fracture risk is due to over-maturation and impaired quality of the bone. They have higher BMD by DXA at all skeletal sites, especially at the lumbar spine [89]. Furthermore, they typically have normal [89,90,91] or low [92] trabecular bone scores and are classified as degraded microarchitecture. Compared to the age and sex-matched controls, they often have higher volumetric BMD (trabecular and cortical), and higher cortical area and thickness by pQCT [89,93]. Nevertheless, HR-pQCT showed increased cortical volumetric BMD, but reduced cortical thickness and cortical porosity [89,94]. They also seem to have normal biomechanical strength determined by finite element modeling [94,95], but lower bone material strength index, measured by impact microindentation, than controls [86,96]. Calcium and vitamin D supplements are widely used. However, the long-term safety and efficacy of this practice are not very well studied. Donovan Tay et al. reported that long-term use of PTH (1-84) therapy reduced supplemental calcium and vitamin D requirements and increased lumbar spine and total hip BMD [97]. PTH (1-84) reduced urinary calcium and serum phosphorus levels and improved quality of life without increasing serious adverse events, compared to traditional management [98,99,100]. In a recent meta-analysis, compared to PTH, active vitamin D usage was associated with similar serum calcium levels but a trend toward lower urinary calcium levels [101]. Moreover, the long-term safety is not completely recognized, and dose-dependent increased risk of osteosarcoma is reported in rat studies [102,103]. This concern limited the long-term usage of PTH (1-84) as replacement therapy for hypoparathyroidism. Small studies reported heterogeneity regarding the efficacy of parathyroid tissue allotransplantation for treating hypoparathyroidism [104]. 

Primary hyperparathyroidism (PHPT) is associated with decreased BMD and increased fracture risk across various skeletal sites, especially at the lumbar spines [105,106]. BMD measurement by DXA is an acceptable predictor of fracture at hip and forearm but underdiagnoses vertebral fragility [107]. There are valuable tools, such as trabecular bone score, 3D-DXA [108], bone strain index (BSI) by finite element analysis of DXA [109], and HR-pQCT [110], to assess bone health and predict skeletal fragility [105]. HR-pQCT revealed altered microarchitecture of cortical and trabecular bone, including reduced cortical and trabecular volumetric densities, increased cortical porosity, and heterogeneity of trabecular distributions [110,111]. This is almost consistent with histomorphometric studies, except for preservation or even improvement of trabecular bone structure [112]. The assessment of bone material strength index at the tibia by using the impact microindentation technique showed impaired bone material properties in PHPT subjects, especially in those with fragility fracture [113]. Parathyroidectomy reduces calcium concentrations and increases BMD at different skeletal sites. It might reduce fracture risk better than active surveillance [114], but its advantages over medical therapy regarding risk of fracture, kidney stones and quality of life lack sufficient evidence [114,115]. Nevertheless, parathyroidectomy could improve bone strength assessed by HR-pQCT and finite element analysis [116]. In terms of medical therapy, optimization of calcium and vitamin D intake is suggested [117]. Calcium supplements can reduce PTH and increase femoral neck BMD in patients with asymptomatic PHPT [118]. There is no reason to restrict dietary calcium intake in the patients with mild PHPT, but close monitoring of calcium is necessary and calcium supplementation should be avoided in severe PHPT with elevated 1,25(OH)2D and higher serum PTH levels. Other medical therapies include bisphosphonates, cinacalcet, denosumab, and estrogen, which are appropriate for lowering calcium, increasing BMD or both [117]. 

### 3.4. Thyroid Disorders

Thyroid hormones play a pivotal role in bone metabolism. Hyperthyroidism, even subclinical, is a known risk factor for osteoporosis. It is associated with increased bone turnover, decreased bone mass, and increased fracture risk [119,120]. In addition, long-term TSH suppression in patients with differentiated thyroid cancer was associated with lower BMD in postmenopausal women [121]. Hyperthyroid women had impaired bone quality and quantity reported by HR-pQCT. Euthyroidism could improve volumetric BMD and cortical microarchitecture [119]. Overt hypothyroidism reduces bone formation. However, data on BMD and fracture risk are inconclusive [122]. 

### 3.5. Adrenal Disorders 

Osteoporosis happens in 30–50% [123,124,125], and vertebral fractures in 30–70%, of patients with Cushing syndrome [126,127]. Cushing syndrome leads to excess glucocorticoid production, which negatively impacts bone metabolism through suppression of growth hormone and gonadal axis, besides altering the rhythmic production of parathyroid hormone [126]. Trabecular bone loss is more pronounced in patients with Cushing syndrome. Adrenal nodules with autonomous cortisol secretion [128], primary aldosteronism [129], pheochromocytoma [130,131], and congenital adrenal hyperplasia [132] are associated with deterioration of bone quality and quantity. 

### 3.6. Growth Hormone 

Despite acromegalic patients having a higher rate of bone formation, they have an increased risk of vertebral fractures because of increased bone turnover and poor bone quality. However, they may have increased, decreased, or similar BMD, compared to the general population [133,134]. They have higher cortical porosity and altered bone microarchitecture, which is attributed to altered bone remodeling and Wnt signaling.

Growth hormone deficiency is associated with low bone turnover osteoporosis, and loss of cortical greater than trabecular bone, which leads to increased fracture risks [135]. Growth hormone replacement initially increases bone turnover and reduces bone density. A maintenance treatment encourages improved bone mass, but its effects on fracture risk is not definite [136]. This could be due to increased DKK-1, a Wnt inhibitor, therefore increasing cortical porosity [137]. 

## 4. Gastrointestinal Causes

Malabsorption and chronic liver disease are well-known causes of osteoporosis, and they are included in the FRAX. Physiologic bone metabolism requires optimum amounts of nutrients, particularly minerals and vitamins. Vitamin D is a fat-soluble vitamin, so bone loss is remarkable in diseases associated with fat malabsorption [138,139,140,141,142,143]. Furthermore, in cases of steatorrhea, calcium absorption may be hindered by binding to excess fatty acids in the gastrointestinal (GI) lumen [144]. In this section, we will discuss the most common causes of GI-related osteoporosis.

### 4.1. Celiac Disease 

Patients with celiac disease have a high prevalence of osteopenia and osteoporosis, 40% and 15%, respectively, even after excluding postmenopausal women [145]. It has been reported that 8% of patients with idiopathic low BMD have positive IgA anti-endomysial antibodies even though they are asymptomatic. Routine screening for celiac disease might be considered in idiopathic cases of osteoporosis [146,147]. A gluten-free diet can significantly improve BMD [148,149]. However, bone loss may persist due to the continuous inflammatory process leading to higher osteoclast activity and lower ability to generate bone matrix [150].

### 4.2. Chronic Pancreatitis 

More than 50% of patients with chronic pancreatitis, particularly smokers and alcoholics, have low BMD. Pancreatic enzymes and vitamin D replacement significantly lowered the risk of fracture [151]. Cystic fibrosis can disturb bone health through mechanisms other than malabsorption. Cystic fibrosis transmembrane conductance regulator is expressed in bone cells, thus it might have a negative impact on bone metabolism. Additionally, bone resorption increases during pulmonary exacerbations as the proinflammatory cytokines stimulate osteoclast activity [152]. 

### 4.3. Short Bowel Syndrome

The prevalence of osteoporosis in patients with short bowel syndrome is 2-fold higher compared to matched controls [141]. Bone loss occurs because of micro and macronutrients’ malabsorption. Metabolic acidosis, either caused by chronic diarrhea or D-lactic acidosis by bacterial overgrowth, can also impair bone health [153].

### 4.4. Hepatic Osteodystrophy

Disturbed enterohepatic circulation of fat-soluble vitamins impairs bone metabolism. This is one of the main causes of bone loss in biliary disorders as primary biliary cholangitis (PBC) and sclerosing cholangitis. The prevalence of osteoporosis and fractures in PBC is up to 50% and 20%, respectively [154,155,156]. The etiology of chronic liver disease, alcohol, viral hepatitis, and autoimmune diseases, may contribute to the pathogenesis of hepatic osteodystrophy [154,157,158,159,160,161]. Cirrhosis complications such as malnutrition, impaired physical activity, and hypogonadism, along with disturbed vitamin D and K metabolism [162,163], can aggravate bone loss.

### 4.5. Peptic Ulcer Disease

Peptic ulcer disease is linked to osteoporosis, especially among males. Certain species of H-pylori infections may afflict bone metabolism by enhancing inflammatory status, reducing the circulatory ghrelin and estrogen levels, and increasing postprandial serotonin levels. Moreover, long-term use of PPIs can impair bone health [164,165].

### 4.6. Inflammatory Bowel Disease (IBD)

Patients with IBD have a higher risk of bone loss [166], poor bone quality [167,168], and fractures [169,170,171,172]. This can be explained by malnutrition, chronic inflammatory process, and immunosuppressive drugs [171,173,174]. Low turnover bone disease is the predominant underlying pathology in patients with osteoporosis and IBD [175,176]. The American College of Gastroenterology recommended using the conventional risk factors as indications for BMD screening in IBD patients using DXA scan [177]. The Cornerstone Health organization has expanded the indications for BMD screening to include maternal history of osteoporosis, malnourished or very thin patients, and amenorrheicor postmenopausal women [178]. Maldonado and colleagues highlighted the role of biomechanical CT to detect patients with an increased risk of fracture. 40% of those patients were not included in the Cornerstone checklist. Thus, IBD patients undergoing CT enterography may benefit from biomechanical CT screening for fracture risk [179]. Early suppression of the inflammatory process by anti-TNF is associated with better bone preservation [169,180]. In addition to calcium and vitamin D optimization, bisphosphonates are relatively safe and effective treatment options [181]. In an animal study, a natural compound (emodin) has been reported to inhibit osteoclast function and prevent IBD-related osteoporosis [182].

### 4.7. Irritable Bowel Syndrome

Patients with irritable bowel syndrome have a higher incidence of osteoporosis and fragility fractures [183]. This might be explained by chronic inflammation, overactivation of the hypothalamic-pituitary-adrenal axis, nutritional deficiency, and smoking. Further studies are needed to confirm the underlying mechanisms and to establish a treatment approach [184]. 

### 4.8. Dysbiosis 

Microbiota is considered a hidden organ that has a bidirectional interaction with cellular responses. Certain species of microbiota are linked to osteoporosis and autoimmune diseases such as IBD, PBC, and sclerosing cholangitis [185,186]. The beneficial effects of probiotics were explained experimentally by manipulating the expression of OPG/RANKL, Wnt10b, and inflammatory cytokines [186,187]. 

Other GI disorders with increased risk of osteoporosis include post-gastrectomy [188], atrophic gastritis [189,190], and bariatric surgeries [191].

## 5. Nutritional Causes

Nutritional factors can potentially affect bone mass, metabolism, matrix, and microarchitecture. Insufficient nutrition leads to deficiency of protein, vitamins, and minerals, particularly calcium, phosphorus, and magnesium, which are essential for bone health [192]. The recommended daily calcium intake for adults is between 800–1200 mg daily [32,193], while it is 700 mg and 320–420 mg for phosphorus and magnesium, respectively [194]. The daily protein requirement is recommended to be 0.8 gm/kg for adults and 1–1.2 gm/kg for the elderly [195,196]. The vitamin D daily requirement ranges from 800 to 1000 IU [197]. 

Malnutrition can happen either because of poor nutrient intake, increased losses, and/or increased demand [198]. Bad dietary habits, anorexia nervosa, bulimia nervosa, prolonged total parental nutrition (TPN), bariatric interventions, and excess alcohol intake can cause secondary osteoporosis [199]. As osteoporosis and fractures are associated with many life-threatening events, their prevention is essential via balanced diet and physical exercise [200]. 

Starvation, the most severe form of malnutrition, can be caused by various socio-economic, environmental, and medical factors [201]. Starvation can negatively affect bone quantity and quality through minerals, vitamins, and collagen type I deficiency [201,202]. There is a positive relationship between malnutrition during early life, or even in utero, and early incidence of osteoporosis and fractures [203,204,205,206,207]. 

Vitamin D deficiency results in decreased calcium absorption and hypocalcemia leading to secondary hyperparathyroidism, consequently stimulating bone turnover and decreased BMD [208]. Treatment with vitamin D supplements has beneficial effects on bone health in patients with 25-hydroxy vitamin D levels less than 30 nmol/L [209,210]. On the other hand, prophylactic doses of vitamin D have a debatable role in the prevention of osteoporosis and fractures. [211,212,213,214,215,216].

Many observational studies reported a positive relationship between body mass index (BMI) and BMD [217]. Moreover, previous studies demonstrated that obesity could protect against fractures [218,219]. However, more recent studies did not show a positive impact of obesity on bone [220]. The Look AHEAD trial reported a modest increase in bone loss at the hip with intensive non-surgical weight loss interventions in obese type 2 diabetics [221,222]. Moreover, most bariatric surgeries were associated with bone loss and fragility [191,223]. This may be explained by mechanical unloading, secondary hyperparathyroidism due to malabsorption of calcium and vitamin D, decreased estrogen, leptin, and ghrelin, and increased adiponectin levels [191,224,225]. Therefore, it is recommended to receive adequate calcium and vitamin D and to monitor BMD after bariatric surgeries [226]. 

Patients with anorexia nervosa extremely limit their food intake because they are scared of weight gain [227]. This can lead to several medical complications including bone loss [228] with a 2–7-fold increased risk of fractures [229,230]. This is not only because of nutritional deficiencies but hormonal disturbances as well [231]. On the other hand, improving nutritional status corrects the endocrinological disorders and BMD in these patients [232]. Anti-osteoporotic medications may help to ameliorate bone loss in patients with persistently low BMI and amenorrhea [233]. Residronate use, either alone or combined with transdermal testosterone, resulted in improved spinal BMD [234,235]. Moreover, physiological doses of transdermal estrogen lead to increased spinal and hip BMD [236]. In a recent RCT, sequential therapy with recombinant human IGF-1 and risedronate was superior to risedronate alone in improving lumbar spine BMD in women with anorexia nervosa [237]. Furthermore, Fazeli et al. reported a significant increase in lumbar spine BMD after 6 months of teriparatide use [238]. 

Patients with prolonged TPN have an osteoporosis prevalence of 40 to 100% [239,240,241]. Despite TPN improving nutritional status, the prolonged need for TPN may induce dysbiosis [242], decreased gut calcium, and phosphorus absorption [239]. Moreover, it can induce hypercalciuria because of the hyperfiltration secondary to high amino acid infusion [243]. Routine vitamin D monitoring and management are necessary for patients with prolonged TPN because vitamin D deficiency is very prevalent among these patients [239]. Bisphosphonates improved BMD in patients with TPN-associated osteoporosis [244,245]. 

Bad dietary habits have been reported to be associated with osteoporosis. High dietary sugar may lead to osteoporosis [246] by glucose-induced hypercalciuria, hypermagnesuria [247,248], and decreasing vitamin D activation [249]. In addition, hyperglycemia can decrease osteoblast proliferation and increase osteoclast activation [250,251]. On the other hand, the effect of dietary salt on bone health is unclear [252]. 

Heavy alcohol intake has been associated with decreased BMD [253]. Mechanistically, it directly reduces osteoblast activity and increases osteoclastogenesis [254,255,256]. Indirectly, it can cause changes in body composition [257] and alterations in various hormones, including PTH, vitamin D, testosterone, and cortisol [258]. Alcohol abstinence may improve bone metabolism and increase BMD [259,260].

## 6. Drug-Induced

Drug-induced osteoporosis is the second most common cause of secondary osteoporosis. Despite their well-known adverse events, glucocorticoids are still one of the cornerstone immune-suppressive/modulator and anti-inflammatory therapies. Up to 40% of patients on long-term glucocorticoid therapy suffer from fractures during their lifetime [261,262].

Areas with high trabecular bone, such as lumbar spine and hip trochanter, are the classic sites for glucocorticoid-induced fractures [263]. Robust bone loss may reach up to 20% within the first year of therapy, and subsequently decline to 1 to 3% annually [264,265]. The fracture risk with glucocorticoid therapy is dose and time-dependent [262]. The impact of glucocorticoids on bone has been linked to their cumulative effect, which disturbs both bone quantity and quality. Glucocorticoids can induce bone loss irrespective of the route of administration. For instance, long-term inhaled glucocorticoids were associated with a 10% loss of BMD [266,267]. Even controlled-release budesonide and topical corticosteroid can negatively impact bone health [268,269].

Glucocorticoids initially decrease bone formation and increase RANKL/osteoprotegerin ratio, inducing high bone resorption [270,271]. The mechanism of bone loss with long-term usage is more attributed to suppressed bone formation rather than increased bone resorption. This could be due to the downregulation of the Wnt signaling pathway which impairs the osteoblast activity [272]. Additionally, glucocorticoids have an indirect impact on bone through their effects on calcium homeostasis, parathyroid gland activities, and vitamin D metabolism [273,274]. Furthermore, glucocorticoids lead to loss of muscle mass and strength which increases the risk of falls and fractures. They can also induce hypogonadism which decreases the anti-resorptive effect of testosterone and/or estrogen [275]. 

The use of a DXA scan and FRAX after 6 months of glucocorticoid therapy is recommended for those with a history of fragility fracture, patients of 40 years of age or older, and those with major osteoporotic risk factors [276].

For prevention of glucocorticoid-induced osteoporosis, daily intake of 1000–1200 mg calcium and 600–800 units of vitamin D, along with lifestyle modification, are highly recommended [275]. For adults with high risk of fracture, treatment with oral bisphosphonate is the preferred line of therapy [276]. Teriparatide is also effective in preventing and treating glucocorticoid-induced bone loss [277]. 

Antidepressants like selective serotonin reuptake inhibitors and monoamine oxidase inhibitors can induce low bone density and increase incidence of fracture [278,279,280,281]. It is not clear how these medications affect bone health, but it may be attributed to diminished osteoblast proliferation through the serotonin receptors and transporters [282].

Many studies showed significant bone loss with long-term use of antiepileptic drugs [283,284,285]. The pathogenesis is multifactorial, however accelerated vitamin D metabolism is a crucial co-player [286,287,288,289]. Bone loss occurs as a result of bone remodeling abnormalities rather than abnormal mineralization [290,291,292].

Aromatase inhibitors, adjuvant long-term therapies for breast cancer, lead to abrupt deprivation of estrogen and consequently, bone loss [293]. Moreover, concomitant use of gonadotropin-releasing hormone agonists induces up to 7% annual BMD loss [294]. The use of gonadotropin-releasing hormone agonists in prostate cancer patients is associated with increased fracture risk [295,296,297].

Antidiabetic medications can impact bone health either positively or negatively. Peroxisome proliferator-activated receptor gamma (PPARγ) plays an important role in the regulation of bone formation and energy metabolism, along with insulin sensitivity [298,299]. Its stimulation by thiazolidinediones induces bone resorption and inhibits bone formation [300]. Thiazolidinediones decreased the BMD and increased the risk of osteoporosis when compared to other anti-diabetic medications [301]. The effects of sodium-glucose cotransporter-2 (SGLT2) inhibitors on bone metabolism and fracture risk are receiving more attention because of their wide use. They may increase bone turnover, disturb bone microarchitecture, and reduce BMD [302]. In a recent study, Koshizaka and colleagues reported increased TRAP 5b with no change in BMD in a 24-week RCT [303]. 

In 2010, the FDA released a warning against long-term use of proton pump inhibitors (PPIs) as it may increase the incidence of osteoporosis and fracture risk [304]. The limited available evidence suggested that this might happen through histamine over-secretion [305], and affecting mineral homeostasis [306,307]. There is inconsistent data regarding the impact of PPIs on BMD.

Despite the negative effect of anticoagulants on bone metabolism having been studied for a long time, such effect is still debatable, and the underlying mechanisms are still poorly understood [308]. Unfractionated heparin was associated with significant bone loss compared to low molecular weight heparin [309,310,311]. Long-term use of warfarin was associated with decreased BMD and TBS [312]. In a recent study, this negative effect on bone was more pronounced in warfarin but was also found in direct oral anticoagulants [313]. 

## 7. Infection

Chronic active infections are not infrequent causes of bone loss, mainly due to cytokine release that stimulates osteoclastogenesis and suppresses osteoblast function. Human immunodeficiency virus (HIV)-infected patients have a three times higher prevalence of osteoporosis and up to four-fold increased risk of fractures compared to the general population [314,315]. This might be directly attributed to the HIV infection or secondary to the use of antiretroviral therapy (ART), concomitant alcohol use, smoking, associated hypogonadism, malnutrition, hepatitis B and/or C co-infection, and vitamin D insufficiency [316,317,318,319,320]. HIV infection promotes osteoblast apoptosis and osteoclast activation [321,322,323,324,325]. Furthermore, HIV infection induces a state of chronic inflammation in addition to immune system activation afflicting bone health [326,327,328]. Tenofovir disoproxil fumarate (TDF) is associated with osteoporosis and fractures more than the newer ART [329,330,331,332], as it induces multiple renal tubular defects and mineral losses [333,334]. The European AIDS Clinical Society (EACS) [335] guidelines recommend tenofovir alafenamide (TAF) as a first-line therapy instead of TDF in patients with progressive osteopenia or osteoporosis, as it is less toxic to the renal tubules [336,337]. Bisphosphonates are used effectively for the treatment of HIV-related bone disease [338]; however, bone anabolic drugs have not been adequately studied [315].

Hepatitis B and C viral (HBV; HCV) infections even without subsequent liver cirrhosis is associated with an increased risk of osteopenia and osteoporosis [158,339,340,341]. Furthermore, previous studies reported that the risk of osteoporosis was still higher in patients with HBV and HCV infections even after adjustment for other osteoporosis risk factors [158,342]. Of note, previous studies reported an increased risk of fractures in patients with HIV and HCV co-infection compared with HIV-infected patients [319]. Interestingly, HCV clearance led to a two-thirds reduction in fracture risk in postmenopausal women with osteoporosis [343].

Herpes zoster infection is associated with osteoporosis [344,345]. This negative effect on bone health may be due to the upregulation of inflammatory cytokines, especially in patients with post-herpetic neuralgia [346,347]. 

COVID-19 might predispose patients to osteoporosis [348]. This may be because of associated pro-inflammatory cytokine production and prolonged immobilization in severe cases [349]. Furthermore, there might be direct sequelae of infection on the skeleton [350]. The virus can decrease ACE2 expression in both osteoblasts and osteoclasts [351], leading to disordered bone formation and resorption. In addition, corticosteroids used in the treatment of COVID-19 have a negative impact on bone.

Osteomyelitis is commonly associated with significant bone loss and subsequent fragility fractures [352]. This is mainly attributed to the upregulation of inflammatory cytokines such as IL-1, IL-6, and TNFα with subsequent activation of RANKL and inhibition of osteoprotegerin [353,354].

Patients with active TB and TB survivors with pulmonary fibrosis have increased risk of osteoporosis [342,355]. Chronic systemic inflammation, concomitant malnutrition, and vitamin D deficiency are the main contributors to bone loss [354,356,357,358]. 

## 8. Hemato-Oncological Causes

Hematologic disorders are potentially able to damage bone through direct cellular effects or indirectly, mediated by several circulating factors [359]. The bone loss occurs mainly due to an imbalance between RANKL/RANK and WNT signaling pathways with subsequently increased bone resorption and decreased bone formation [360,361,362,363].

Anemia can lead to bone resorption and increases bone fragility [364,365]. Iron deficiency may negatively impact cytochromes’ P450 activity, which is essential for vitamin D metabolism and bone health [366]. β thalassemia causes ineffective erythropoiesis and bone marrow expansion that leads to medullary destruction and cortical thinning [367]. Moreover, pubertal delay, cytokine disturbances, growth hormone deficiency, iron bone deposition, deferoxamine-induced bone dysplasia, and vitamin D deficiency can further contribute to inadequate bone health in thalassemia patients [368,369,370,371,372,373]. Bisphosphonate may improve BMD [373,374], however its effect on fracture rate is uncertain in patients with thalassemia [375]. There is limited information but promising results observed by using denosumab or teriparatide to increase bone density in thalassemia patients [376,377].

The estimated prevalence of secondary osteoporosis in hemophilia patients is up to 58.7% [378]. The underlying mechanisms for low bone mass include vitamin D deficiency, limited physical activity secondary to hemophilic arthropathy, and the acquisition of osteoporosis-linked blood-born infections such as HIV [379,380,381]. In addition, Factor VIII deficiency is directly associated with increased bone resorption and decreased formation secondary to the imbalance in OPG/RANK/RANKL system [382,383]. Screening for osteoporosis should be implemented in the underweight, those with fragility fractures, HIV, and/or advanced hemophilic arthropathy [384]. Replacement of the deficient factor could minimize joint bleeding and hemoarthropathy and subsequently reduce the risk and progression of osteoporosis [385].

Both monoclonal gammopathy of undetermined significance and multiple myeloma patients are at increased hazard for osteoporosis and fragility fractures [386,387]. Myeloma cells stimulate the release of cytokines, IL-6, and IL-7, leading to activation of the RANKL/RANK pathway and enhanced bone resorption [361]. On the other hand, the expression of WNT inhibitors, Dkk-1, and secreted frizzled protein-2 is enhanced, leading to reduced bone formation [388,389]. Several guidelines recommend myeloma screening in elderly patients with osteoporosis and/or fragility fractures [390,391]. Bisphosphonate is recommended in myeloma patients as they have antineoplastic, immunomodulatory, and anticatabolic effects [392,393]. Nevertheless, renal impairment which is frequent in those patients is still an important hindrance [394] and may obligate the use of other safer drugs such as denosumab [395]. In addition, treatment of multiple myeloma, such as bortezomib, and monoclonal antibodies targeting DKK1 or sclerostin can reduce bone loss [396,397,398].

Osteoporosis is the most common skeletal pathology that occurs in 18 to 40% of systemic mastocytosis patients [399,400,401]. Bone involvement occurs due to bone marrow infiltration by mast cells, besides the release of circulating factors, such as histamine, prostaglandins, and interleukins (IL-1, IL-3, IL-6), which enhance osteoclast activity [402]. The manifestations consist of a wide clinical spectrum from asymptomatic condition to varying degrees of bone damage, such as osteopenia, osteoporosis, osteolytic lesions, and osteosclerosis [403]. Besides antiresorptive medications such as bisphosphonate and denosumab [404,405], interferon may improve bone pathology by controlling the disease activity [406]. Contrarily, safety concerns exist with the use of teriparatide as it may enhance the proliferation of malignant cells [407].

In patients with solid tumors, bone damage usually occurs either as a side effect of anticancer treatment or secondary to osteolytic metastasis, most commonly from breast cancer [408]. Moreover, cytotoxic chemotherapy and hormone deprivation therapies have detrimental effects on both bone quantity and quality [409,410,411]. Bone loss in patients receiving aromatase inhibitors or androgen deprivation therapy is up to ten times that in age-matched healthy controls [412,413]. Therefore, baseline and follow-up DXA scans are serially recommended based on the underlying diseases [414,415]. Bisphosphonate is advised in aromatase inhibitor receivers with higher fracture risk [416].

## 9. Rheumatological-Immunological Causes

The immune system plays an important role in bone homeostasis. Activated T cells affect bone health through the secretion of various cytokines [417]. Some experimental studies detected that Th17 cells are responsible for stimulating bone resorption, while T reg cells are peculiarly associated with inhibition of bone resorption [418]. Moreover, CD8+ T cells might have a protective function through the secretion of various factors, such as osteoprotegerin and interferon-γ which have an anti-osteoclastogenesis effect [419].

### 9.1. Inflammatory Arthritis

Inflammatory arthritis, including rheumatoid arthritis (RA), psoriatic arthritis, and spondyloarthropathy, is frequently associated with systemic skeletal complications, such as osteoporosis and fragility fractures [420]. 

Osteoporosis prevalence in patients with RA is about 30% and increases up to 50% in post-menopausal women [421,422]. Furthermore, a large meta-analysis revealed that patients with RA have a higher risk of fracture [423]. 

RA-related osteoporosis is described by two main features: local and systemic bone loss [424]. Several mechanisms are involved in the pathogenesis of bone loss including sustained inflammation, glucocorticoid use, decreased physical activity and increased secretion of proinflammatory cytokines such as IL-6, IL-1, and TNF-α [422,425]. Moreover, overexpression of RANKL promotes osteoclastogenesis [426]. There is enough evidence to support the role of autoantibodies in the pathogenesis of both local and systemic bone loss through osteoclast activation [427,428,429].

Disease-modifying anti-rheumatic drugs (DMARDs) don’t only control the inflammatory status but also help to avoid the long-term negative effects of corticosteroids on bone health [430]. The use of leflunomide was associated with a significant increase in lumbar spine BMD [431]. Moreover, TNF-inhibitors improved BMD and reduced the rate of fracture [432]. Other biological agents such as tocilizumab, rituximab, and abatacept significantly reduced bone resorption markers and RANKL expression [433,434]. On the other hand, the impact of methotrexate on bone loss is controversial [435].

Despite several studies demonstrating a significant association between psoriatic arthritis and bone loss/fragility fracture [436,437], others did not find such association [438]. Pro-inflammatory cytokines are involved in the mechanism of local bone loss [439].

On the other hand, patients with ankylosing spondylitis (AS) have lower BMD, even in the early stage of the disease [440]. The prevalence of osteopenia and osteoporosis is about 54% and 16%, respectively, within 10 years of the disease onset [440]. A large database showed a higher risk of vertebral and non-vertebral fractures among patients with AS [441]. The use of non-steroidal anti-inflammatory drugs was associated with decreased fracture risk [441]. TNF-inhibitors increased lumbar spine and total hip BMD, however they did not decrease the rate of vertebral fractures [442].

### 9.2. Systemic Lupus Erythematosus (SLE)

Osteoporosis and fragility fractures are well-known comorbidities in patients with SLE [443]. The incidence of fracture is up to 35% in this patient population [444]. Furthermore, asymptomatic vertebral, and non-vertebral fractures were associated with decreased quality of life and increased risk of mortality [445,446]. Pro-inflammatory cytokines directly affect bone mass [447]. Organ damage can indirectly cause bone mass loss. The disease duration and severity, besides the long-term glucocorticoid usage, are the main determinants of bone loss [448,449]. Lower levels of P1NP are predictive of bone loss and decrease BMD over 12 months in premenopausal SLE patients [450].

### 9.3. Multiple Sclerosis (MS)

Several studies showed that people with MS have lower BMD, higher rates of osteoporosis, and increased fracture risk compared to healthy controls [451,452,453]. Various risk factors contribute to bone loss in patients with MS, including disease duration and severity, vitamin D insufficiency, cumulative steroid dose, decreased ambulation, and inflammatory processes [453,454]. The pro-inflammatory osteopontin levels increase in patients with MS and correlate with femur neck BMD [455].

## 10. Others 

### 10.1. Smoking

Smoking is incorporated within the FRAX score as a risk factor for osteoporosis [456]. It has direct harmful effects on osteogenesis and bone blood flow [457]. Indirectly, it afflicts serum levels of vitamin D, PTH [458,459], and sex hormones, particularly in females [460]. The effect of smoking cessation on BMD is unclear; however, it has been shown that it could increase BMD at femur and total hip [461] and reduce vertebral fractures [462]. 

### 10.2. Disuse Osteoporosis

Osteocytes have certain mechano-receptors that use weightbearing-induced signals to orchestrate bone turnover. Immobility leads to osteocyte dysfunction and subsequent inhibition of bone formation via downregulation of Wnt/β-catenin pathway [463]. This can be a systemic disorder with prolonged immobilization or a local disease among patients with hemiparesis, spinal cord injuries, or neuromuscular diseases. Physical exercise and rehabilitation programs are essential in preventing and treating this type of bone loss. The use of antiosteoporotic medications such as bisphosphonates, denosumab, teriparatide, and romosozumab might be indicated in refractory cases [463,464,465]. 

### 10.3. Genetic Causes of Osteoporosis 

Genetics plays a crucial role in bone microarchitectural properties, skeletal strength, and the risk of osteoporosis. Rare, monogenic forms of osteoporosis start in childhood or young adulthood [466]. The most common one is osteogenesis imperfecta (OI), also known as ‘brittle bone disease’ [467]. Osteogenesis imperfecta is a genetic connective tissue disorder caused by defective bone formation, mainly due to impaired production and/or processing of type 1 collagen [468]. It is characterized by an abnormally high bone matrix mineralization. This is related to a larger number of crystals with the same volume of matrix [469,470]. Cortical porosity, thin trabeculae, abnormal bone quality, and low bone density with associated increased risk of fracture are common findings in OI [471,472,473]. There is limited evidence that bisphosphonates increase BMD and decrease the risk of fracture in patients with OI [474]. Moreover, denosumab had poor and inconclusive results [475]. Notably, romosuzumab increased BMD and improved turnover biomarkers in those patients [419]. Apart from OI, whole-genome sequencing studies were able to unmask new genetic variants that are associated with osteoporosis. The expression of these genetic variants is involved in different bone-protecting functions, such as vitamin D metabolism, mesenchymal stem cell osteogenic differentiation, and bone morphogenetic proteins. Some of these variants are population-specific and others are shared between patients with low BMD from different races [476,477].

Osteoporotic fractures are increasing exponentially [478] and are considered one of the major health care problems [479]. Osteoporosis is associated with a negative effect on fracture healing, especially in unstable and comminuted fractures, which indicate internal fixation [480,481]. The power of screw holding is decreased in the osteoporotic bone which, in turn, causes implant loosening, loss of fixation, and impaired healing. Antiosteoporotic medication should be considered to improve bone formation and the success rate of bone implants in osteoporotic fractures [482].

The current review is limited by the quantity and quality of the clinical studies in this field. Few RCTs demonstrated the impact of different anti-osteoporotic medications on bone.

## 11. Conclusions

Secondary osteoporosis is diagnosed when bone fragility is caused by a disease, drug, or nutritional deficiencies. It is an evolving, devastating health problem. Proper diagnosis and prevention are the cornerstones of preventing further bone loss and fragility fractures. Although causal treatment is essential, antiosteoporotic medications can further decrease the risk of fractures, as well as improve fracture healing. More RCTs are required to explore the safety and efficacy of antiosteoporotic drugs in various clinical settings.

## Data Availability

Not applicable.
